# Efficacy of Plant Tissue Culture Techniques for Eliminating Black Mulberry Idaeovirus (BMIV) from Infected Black Mulberry (*Morus nigra*)

**DOI:** 10.3390/plants13212959

**Published:** 2024-10-23

**Authors:** Doaa Waseem Abdelwahab Elansary, Kahraman Gürcan, Vahid Roumi, Özhan Şimşek

**Affiliations:** 1 Genome and Stem Cell Center, Department of Agricultural Biotechnology, Erciyes University, 38280 Kayseri, Türkiye; doaaalansary@gmail.com (D.W.A.E.); vroumi@maragheh.ac.ir (V.R.); 2 Department of Plant and Microbiology, Faculty of Science, Damanhour University, Damanhour 22511, Egypt; 3 Plant Protection Department, Faculty of Agriculture, University of Maragheh, Maragheh 55187, Iran; 4 Department of Horticulture, Erciyes University, 38280 Kayseri, Türkiye; ozhansimsek@erciyes.edu.tr

**Keywords:** mulberry, virus elimination, tissue culture, meristem culture, thermotherapy, chemotherapy, cryotherapy

## Abstract

Obtaining virus-free plants is a crucial step in disease management that enables reliable and profitable fruit farming. The present study applied various in vitro virus elimination protocols, including apical shoot culture, chemotherapy, thermotherapy, cryotherapy, and their combination, to eliminate black mulberry Idaeovirus (BMIV) from sour black mulberry. First, a shoot tip (0.5–2 mm) culture protocol was optimized, and four ribavirin concentrations (0, 10, 20, and 30 mg/L) were investigated over five weeks as a form of chemotherapy (ch). For the first thermotherapy treatment (Ch + Th^1st^), chemotherapy treatment was followed by a gradual increase in the temperature (24–33 °C). In another experiment (Th^2nd^ + Ch), in vitro shoots were incubated in the dark for two weeks at two different temperatures (35 ± 1 °C and 37 ± 1 °C, for one week each). Subsequently, the shoot tips were incubated with various ribavirin doses. Finally, cryotherapy (Cr) was used with or without immersing the shoot tips in liquid nitrogen. A two-step RT-PCR was performed to assess the presence of the virus in 7–8-week-old in vitro plants. Th^2nd^ + Ch significantly increased the shoot tip burst and plant survival/morphogenesis compared to the other treatments. Except for the application of cryotherapy, the protocols eliminated BMIV in different proportions, and the highest virus elimination rate (50%) was obtained by applying 30 mg/L ribavirin during the Ch + Th^1st^ treatment. These findings are essential in preventing the dissemination of the virus and enabling the safe movement of germplasm around the world.

## 1. Introduction

Black mulberry (BM, *Morus nigra* L.) belongs to the Moraceae family, along with 10–16 other species, which also include figs, Osage orange, and banyan [1]. Mulberries are an economically significant crop species grown for their leaves for silkworm (*Bombyx mori*) cultivation. Furthermore, their fruits are consumed as fresh and processed products, such as juices, salads, and dried fruits. Black mulberry plants are highly valued and considered the king of mulberries, mainly due to their fruit taste and pharmaceutical benefits [2,3]. Iran, Anatolia, and the Caucasus are suggested to be the center of origin of this species [1,4]. Black mulberry plants are long-living deciduous trees, 6–9 m in height [5]. Many BM trees are common in Türkiye, believed to have survived for thousands of years, dating back to Roman times and still standing in old towns [4,6].

Like other plants, mulberries host viruses and viroids, including a viroid-like RNA associated with a mosaic dwarf of mulberry [7], hop stunt viroid [8], mulberry vein banding virus (genus *Tospovirus*) [9], mulberry mosaic dwarf-associated virus (genus *Geminivirus*) [10], mulberry mosaic leaf roll-associated virus (genus *Nepovirus*) [11], and fig badnavirus 1 (*Badnavirus fici*) [12]. Recently, high-throughput sequencing technologies have discovered more viruses infecting mulberries [13,14].

A recent study to identify the cause of viral-like symptoms on the leaves of BM trees resulted in the identification of a new virus, tentatively named black mulberry Idaeovirus (BMIV). The symptoms of BMIV infection include deformation, a mosaic look, vein clearing, necrosis of the leaves, deformation, crumbling, and scabs on the fruits [15]. In another study, regardless of whether the trees expressed viral symptoms, all the tested BM trees and tissue culture-propagated plants hosted BMIV, along with mulberry badnavirus 1 (MBV-1) (genus *Badnavirus*) [12]. BMIV and MBV-1 were also detected in pollens and seed-borne black mulberry saplings [15]. According to our recent country-level surveys, all the BM trees tested in Western Asia (*n* = 350) were infected with MBV-1 and BMIV, regardless of symptom presence (unpublished data).

BMIV is a member of the Idaeovirus genus (family *Mayoviridae*) [16], which includes raspberry bushy dwarf virus (RBDV; *Idaeovirus rubi*), which was the first idaeovirus recognized by the International Committee on Taxonomy of Viruses [17]. The genus also includes one confirmed (privet leaf blotch-associated virus; PrLBaV; *Idaeovirus ligustri*) [18] and eight proposed species: citrus idaeovirus (CIV) [19], blackcurrant leaf chlorosis-associated virus (BCLCaV) [20], green Sichuan pepper idaeovirus (GSPIV) [21], birch idaeovirus (BIV) [22], camellia yellow ringspot virus (CaYRSV) [23], Zhuye pepper idaeovirus, Idaeovirus sp., and black mulberry idaeovirus (BMIV) [15]. Idaeoviruses can be transmitted by seeds and pollens [24]. Idaeoviruses account for significant financial losses; in the case of RBDV, 50% fruit loss has been reported [25].

With the advent of tissue culture techniques, virus-free plants can be produced through various methods, including meristem culture, thermotherapy, chemotherapy, and cryotherapy, and mass propagated in the laboratory throughout the year, irrespective of the growing season [26]. These methods for virus-free plant production have been used alone or in combination with other methods to obtain healthy plants for many different plant species, including apples [27,28], pears [29], grapevines [30], hazelnuts [31], quinces [32], and *Narcissus tazetta* [33]. Considering studies on the elimination of Idaeoviruses, almost all of them were aimed at eliminating RBDV. The elimination of RBDV from raspberry (*Rubus idaeus*) plants using conventional thermotherapy and meristem tip culture is challenging because this pollen-transmitted virus efficiently invades leaf primordia and all meristematic tissues, except for the smallest differentiated cells of the apical dome [34,35]. Indeed, traditional thermotherapy and meristem culture resulted in only a few RBDV-free plants [36,37,38]. However, thermotherapy followed by cryotherapy [34] and a combination of chemotherapy, thermotherapy, and cryotherapy [36] have improved the efficiency of RBDV elimination from infected raspberries, as well as eradicated other recalcitrant viruses/viroids in other fruit crops [39,40].

The present study is a pioneering effort in the field, aiming to develop a virus elimination procedure for producing BMIV-free BM plants. It is the first study to eliminate a virus species of the *Idaeovirus* genus other than RBDV and the first to eliminate a virus species in *Morus* plants. Such studies on producing virus-free materials are vital for germplasm conservation and breeding programs.

## 2. Results and Discussion

### 2.1. In Vitro Establishment of Mulberry Accessions

**Figure 1:**
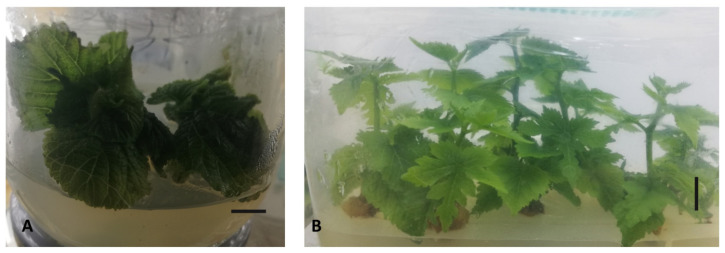
Micropropagation of sour black mulberry (*Morus nigra*) (#695). Initial shoots with big, broad, palmate-type leaves developed from the buds six weeks post-regeneration (**A**), and subcultured shoots with lobed leaves four weeks after regeneration (**B**). Scale bar = 1 cm.

According to our protocol, we successfully regenerated BM plants from an ancient tree named 695 (Figure 1). In the first trials, buds taken at various times (seasons) of the year failed to produce shoots in in vitro cultures. Later, a high rate of shoot development was achieved by peeling the buds. For various studies, especially for micropropagation purposes and virus testing [36], we continuously took buds from mature and young trees, and shoot formation occurred at high rates regardless of the time of year during which the buds were taken (data not presented here). After successful shoot formation (Figure 1), we maintained BM plants in an MS medium with 1 mg/L BA or without hormone supplementation. During the micropropagation of ancient plants, when proliferation was required, we used 1 mg/L BA for increased propagation rates. We also used IBA in the rooting medium in the initial experiments for the rooting phase. However, later, we abandoned the usage of IBA for rooting because the shoots were successfully rooted in MS media without hormone supplementation.

### 2.2. Efficiency of the Treatments

#### 2.2.1. Regeneration Rate

**Figure 2:**
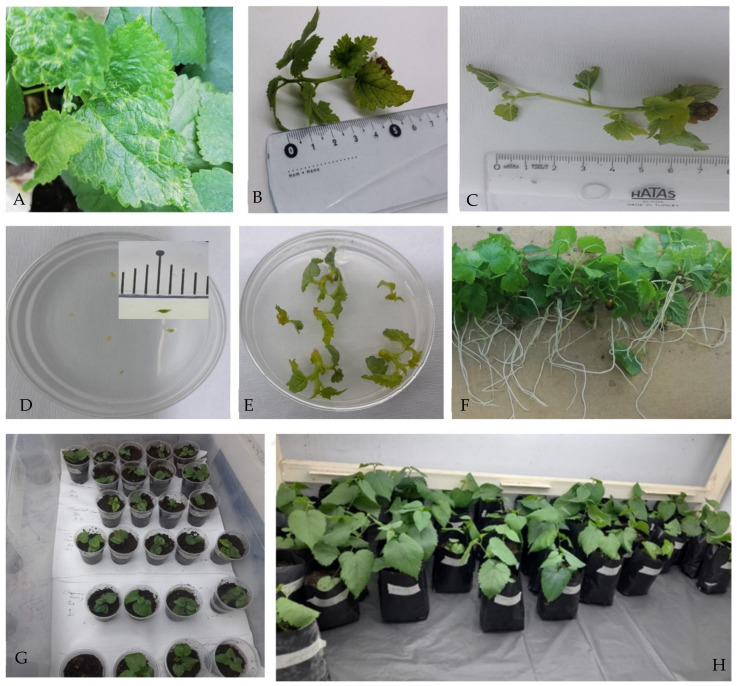
In vitro treatments for eliminating black mulberry idaeovirus (BMIV) applied to sour black mulberry (*Morus nigra*). Viral symptoms on leaves of an ancient tree (#695) used as an infected source (**A**). Enhanced shoot length by thermotherapy (**B**,**C**). Meristem tips (**D**). Five-week-old shoots developed from meristem tips (**E**). Eight-week-old rooted shoots (**F**). In vitro rooted explants sown in peat in 180 mL plastic glasses placed into 80 L transparent plastic baskets for growth and acclimatization at 24 ± 1 °C with a 16 h photoperiod (**G**). Eight-to-ten-week-old BMIV-free plants growing in plastic bags in the climate room (**H**).

A total of 708 shoot tips/meristems were subjected to 17 treatments (Figure 2). There were five controls out of these experiments. After 7–8 weeks, all regenerated explants were ready for subculture. Results showed that cryotherapy provided the lowest survival rate (33.3%), while 10, 20 and 30 mg/L ribavirin treatments conducted after thermotherapy (Th^2nd^ + Ch) provided a 100% survival rate (Table 1). The survival rate for ribavirin treatment alone (58.3–68.3%) was lower than the mock control containing 0 mg/L ribavirin (75%). In the rest of the treatments (Ch + Th^1st^ and Ch + PVS2), different doses of ribavirin led to decreased survival rates compared to the mock controls.

In this study, three doses of ribavirin and the control were applied either alone on the meristematic culture or in combination with the previously mentioned thermotherapy techniques upon the apical shoot tip culture. However, the plant regeneration rate decreased when ribavirin was applied to meristematic culture; there was no significant difference between the treatments and the control. The 30 mg/L ribavirin did not affect the regeneration, but the seedling morphology was severely distorted. The morphologically deformed plantlets grew normally when transferred to growth media (GM). Inhibitory effects and toxicity of ribavirin have been reported previously in several plant propagation procedures to eliminate viruses and/or viroids from infected propagative material [26].

#### 2.2.2. Virus Removal Efficiency

Table 2 lists the results of RT-PCR (Supplementary Figure S1) using eight primer pairs for BMIV detection in 163 randomly selected tissue culture plantlets regenerated from the different treatments. A primer pair amplifying the 1,5-bisphosphate carboxylase chloroplast gene (RBC) of *Prunus persica* was used to evaluate the quality of total RNA and the effectiveness of the detection assay [41]. Twenty virus-free plantlets were identified. All the virus-free plantlets were transferred to the rooting medium, acclimatized, and then kept in greenhouse conditions.

When shoot tips (0.5–2 mm) were cultured for five weeks on medium supplemented with 10 mg/L ribavirin, all plants tested were still BMIV-infected, whereas 20 mg/L and 30 mg/L applications resulted in 20 and 33.3% virus elimination rates. Among the antiviral agents, ribavirin is a highly effective compound which inhibits viral RNA synthesis by interfering with viral polymerase [26,42]. It has been used for plant virus removal in several cases. Plum pox virus was successfully eliminated from plum cv. Bluefree and apricot cv. Hanita without any toxic side effects on the seedlings by using 10 mg ribavirin for 12 weeks [43]. In another study, applying 20 and 25 mg/L ribavirin for 5–30 days successfully eliminated apple chlorotic leaf spot virus (ACLSV) and apple stem grooving virus (ASGV) from the infected in vitro-cultured sand pear with 50 and 61.5% efficiency, respectively [29].

Thermotherapy decreases the movement of the viral particles towards the apical dome, so it plays a role as a virus elimination technique [29]. In vitro thermotherapy following gradual temperature increment (1 °C per day) for acclimatization of ex vitro plants efficiently eliminated the plum pox virus from the apricot cultivar Bebecou [44]. The previously mentioned thermotherapy protocol increased the plant regeneration rate, but all the organs captured high water concentration inside it, causing morphological deformation. In this study, two weeks of thermotherapy followed by shoot tip culture (0.5–2 mm) failed to eliminate BMIV. Nevertheless, its efficacy was improved when it was combined with ribavirin treatments. The highest virus elimination efficiency was observed for the T1st + Ch treatment. Using 0 and 10 mg/L ribavirin, we failed to regenerate any virus-free plant, while using 20 and 30 mg/L ribavirin for five weeks, 30 and 50% of the tested plants were virus-free, respectively.

The thermal sensitivity of some viruses has been reported to be lower than that of the plant cell; accordingly, the plant cells can recover more easily than the viral particles [45]. Thus, in this study, the in vitro shoots were grown inside a dark and hot incubator according to the previously mentioned protocol for faster growth combined with lower virus titer. The shoot tip culture applied after this protocol succeeded in recovering MBV1-free in vitro plantlets with 40% efficiency. Also, 10, 20, and 30 mg/L could regenerate BMIV-free in vitro plantlets with an efficiency of 20%. Additionally, this protocol resulted in obtaining virus-free plants without any morphological deformation. However, it is worth mentioning that low virus titers can limit RT-PCR sensitivity and give false negative results. False negatives can also occur when the viral RNA target sequence is degraded, or reagents of RNA isolation, cDNA construction and RT-PCR are of insufficient quality. In the present study, we have used eight pairs of primers targeting various viral genome regions to minimize the risk of false negative results. Acclimated virus-free plants will be checked six months later to remove possible false negatives at the in vitro stage.

Although the regeneration rate after cryotherapy was 33%, the procedure did not affect the elimination of BMIV under the conditions used in this study. Even when the PVS2-treated shoot tips were cultivated upon the ribavirin doses, no virus-free in vitro shoots were obtained. However, there are several reports that cryotherapy has been successful in some plant–virus combinations, like plum pox virus (PPV) in *Prunus* [46] and two apple viruses [47]. Compared to El-Homosany et al. [48], this study provided an improved LN-treated shoot tip regeneration protocol. In this protocol, the incubation period for the shoot tips upon the pre-culture medium (PCM) in dark conditions was also decreased to only 24 h.

In the present study, a higher percentage of BMIV-free plants was obtained due to a combination of chemotherapy and thermotherapy; indeed, similar to other studies, eradication frequencies varied when combinations of pathogen eradication therapies were applied [26,48]. Variation in eradication frequencies is attributed to host genotypes, the differences in virus morphology, transmission factors in plant cells, and different sensitivity of virus and host to elevated temperatures [26,49]. For example, Farhadi et al. [32] reported success rates ranging from 26% to 100% depending on the varieties they used for virus elimination. In the present study, we used only one genotype, and other genotypes can give different success rates of virus elimination after different therapy applications. Recent reports have shown that cryotherapy cannot eradicate all types of viruses and, in particular, cannot eradicate those that can infect the meristematic cells [50].

## 3. Materials and Methods

### 3.1. Plant Material and Shoot Initiation

**Figure 3:**
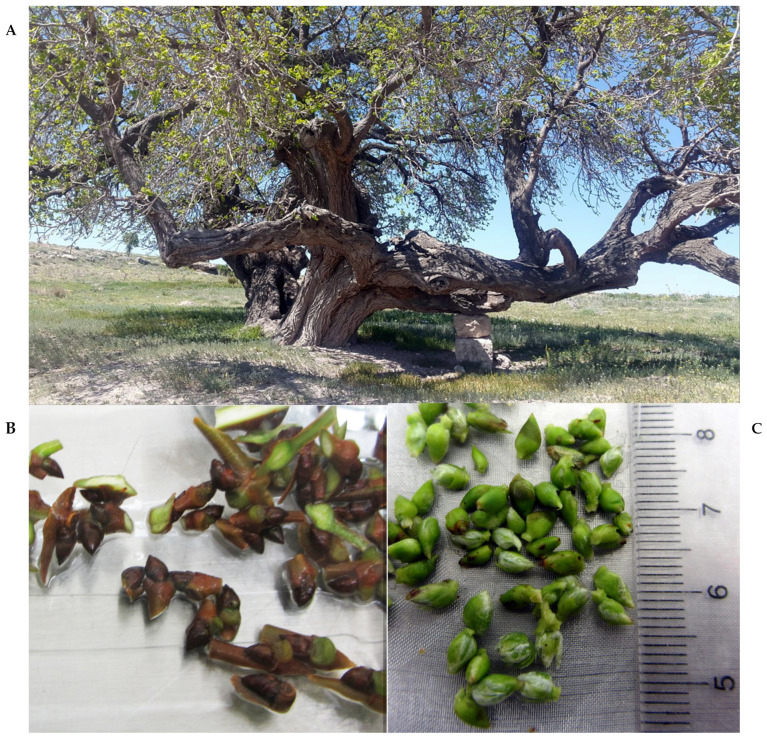
The ancient tree (#695), sour black mulberry (*Morus nigra*), used as a bud source for in vitro initiation (**A**). Buds of the tree (**B**). The buds peeled off for cultivation (**C**).

For the tissue culture experiment, BM buds were harvested from an ancient tree called #695 in a field in Talas, Kayseri, Türkiye (Figure 3A). The presence of BMIV in the tree was determined and verified previously [15]. The tree was chosen as the most promising ancient accession and was therefore used as the source of propagules for the experiments. BM dormant buds about 6 to 8 mm in length were collected from the mature tree in April of 2019 (Figure 1). The buds were surface sterilized in a 2% hypochlorite solution for 15 min. Subsequently, they were washed in double-distilled water several times, and the brown bud bark and one-layer leaflet were removed from the buds (Figure 3B,C). The green buds were again sterilized in a 2% hypochlorite solution for 15 min and rinsed with distilled water. After sterilization, the green buds were cultured in GM, which includes MS basal medium [51] supplemented with vitamins, 3% sucrose, 7 g/L agar, and 1 mg/L 6-benzyl amino purine (BA). The pH of all media was adjusted to 5.8. The maintenance, subculturing, and multiplication of explants were continued using the same media. For micropropagation and plantlet stock maintenance, 60 mL and 40 mL of media were used per gamma-ray-sterilized disposable polystyrene clear wide-mouth tissue culture vessel (Eco2Nv, OV80 + OVD80 with filter “XXL+”, size 150 × 90 × 80 mm) and per 400 mL kitchen-type glass jars, respectively. The temperature of the culture room was maintained at 24 ± 1 °C with a 16 h photoperiod.

### 3.2. Virus Elimination Experiments

#### 3.2.1. Culture Media for Cryotherapy, Thermotherapy, Chemotherapy and Micropropagation

**Figure 4:**
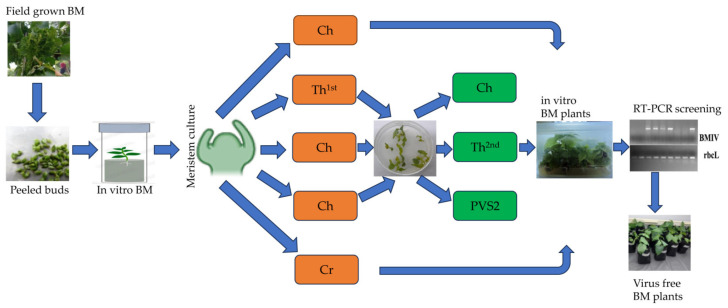
Diagrammatical representation for BMIV elimination treatments applied to sour black mulberry. BM: black mulberry: Ch: chemotherapy; Th: thermotherapy; PVS2: plant vitrification solution 2; Cr: cryotherapy.

All the infected plants used in this study for virus elimination were randomly selected from micro-propagated and maintained BM explants as described above. All the BM in vitro virus elimination processes were conducted using MS, including vitamins. Shoot tips (0.5–2 mm) with 4–6 primordial leaves isolated from 7–8-week-old plantlets growing in GM were used in the experiments. Each experiment included six replicates with six explants each. After each application, explants were transferred to a recovery medium (ReM) consisting of ½ MS + 1 mg/L BA + 3% sucrose solidified with 6.5 g/L agar. The pH of all media was adjusted to 5.8. The experiment’s procedure is presented as a diagram in Figure 4.

#### 3.2.2. Chemotherapy (Ch)

Ribavirin was used as an antiviral compound. It was filter-sterilized by a cellulose filter of 0.22 μm pore size and added into GM at final concentrations of 0, 10, 20, and 30 mg/L. Shoot tips were cultured on 50 mL Petri dishes containing 40 mL of GM with the ribavirin under standard culture conditions for five weeks. After that, explants were removed to ReM.

#### 3.2.3. Thermotherapy after Chemotherapy (Ch + Th^1st^)

After applying chemotherapy to the shoot tips as described above, the vessels containing shoots in ReM were exposed to a gradual thermotherapy application called Th^1st^. For this, first, a gradual increase in the day (16 h) incubation temperature (24 ± 1–33 ± 1 °C) of 1 °C every two days and 1 °C every three days after reaching 30 °C was applied. Afterwards, the temperature remained stable at 33 ± 1 °C as the daytime (16 h) temperature for two weeks, while the nighttime (8 h) temperature was 24 ± 1 °C.

#### 3.2.4. Chemotherapy after Thermotherapy (Th^2nd^ + Ch)

Mother plants at 7–8 weeks old growing in magenta plastic boxes on GM were kept at 35 ± 1 °C for one week and subsequently at 37 ± 1 °C for another week in complete darkness. This thermotherapy application was called Th^2nd^. The shoot tips were then isolated and cultured in the same medium conditions described above for the five-week chemotherapy.

#### 3.2.5. Cryotherapy (Cr)

The shoot tips were first cultured on the pre-culture medium (PCM) consisting of MS and 0.2 M sucrose solidified with 7 g/L agar (pH = 5.8) for 24 h. Subsequently, for 20 min, the explants were kept in 1 mL of a liquid loading solution (LS) consisting of ½ MS + 2 M glycerol + 0.4 M sucrose (pH = 5.8) in 2 mL cryotubes. LS was replaced with 1 mL of chilled filter-sterilized plant vitrification solution (PVS2) [34]. PVS2 was prepared using 0.4 M sucrose, 30% (*w*/*v*) glycerol, 15% (*w*/*v*) ethylene glycol, and 15% (*w*/*v*) dimethyl sulfoxide (DMSO) in liquid MS medium, and then the cryotubes were immersed in an ice bath for 30 min. Then, the PVS2 was replaced with 1 mL of fresh chilled PVS2 and directly immersed in −196 °C liquid nitrogen (LN). Subsequently, the PVS2 was replaced with a recovery solution (RS) consisting of liquid MS and 1.2 M sucrose (pH = 5.8) and kept for 30 min at room temperature. Finally, the RS was discarded from the cryotubes, and the explants were dried with sterile filter papers and cultured for two weeks upon 50 mL Petri dishes containing 40 mL recovery medium (ReM) consisting of ½ MS + 1 mg/L BA before they were transferred to GM. The pH of all media was adjusted to 5.8.

#### 3.2.6. Chemotherapy after PVS2 (Ch + PVS2)

The cryotherapy protocol was performed until the LN application stage. Then, the shoots in PVS2 were not immersed in LN but directly cultured upon 50 mL Petri dishes containing 40 mL GM with different concentrations of ribavirin (0, 10, 20, 30 mg/L) for five weeks.

### 3.3. Rooting and Acclimatization of Explants

The explants kept in ReM following the treatments described above were transferred to MS media without a growth regulator for rooting. The 7–8-week-old explants were transferred into turf soil in 180 mL plastic glasses placed into 80 L transparent plastic baskets for growth and acclimatization in a growth room at 24 ± 1 °C with a 16 h photoperiod (Figure 2G). After about six weeks of maintenance in the basket, the basket lid was opened gradually when the explants reached 10 cm in length. On the first day, an opening of 1 cm was left from the short side of the rectangular baskets. The opening was increased by 1 cm each on the next two days. On the fourth day, the lid was removed entirely. Then, the plants were transferred into two litter plant growth bags containing peat. The plants in the growth bag were kept at 24 ± 1 °C with a 16 h photoperiod in the growth room.

### 3.4. Experimental Design and Statistical Analysis

Except for Cr, which consisted of only one treatment, the other experiments comprised four treatments, one for each ribavirin concentration. The experiment had six replicates per treatment; except for chemotherapy treatments, there were eleven replicates. Also, 0 mg/L ribavirin treatment in the Ch. + PVS2 protocol comprised seven replicates. Each replicate was represented with a 50 mL small Petri dish containing 40 mL of media. Six explants were cultivated on each Petri dish. After two weeks, the cultivation media were replaced with fresh media for all the experiments. Also, after five weeks of growth, survival rates at each Petri dish were recorded, and the explants were transferred to small autoclave-sterilized glass jars containing 60 mL of media.

The data were subjected to variance analysis, and the means were compared using Duncan’s multiple range test by SPSS 25 version, and *p* < 0.05 was considered statistically significant.

### 3.5. Virus Indexing by RT-PCR

Eight to ten in vitro shoots were randomly selected for virus screening by RT-PCR from each treatment. The plantlets were sampled after six to nine weeks in the post-regeneration culture when they were about 2 cm long and had 4–6 leaves. The RNA was isolated from 100 mg leaf tissue using RiboEx RNA Extraction Solution (GeneAll, Seoul, Republic of Korea). cDNA was synthesized by random hexamer (Invitrogen, Burlington, ON, Canada) using Moloney murine leukemia virus (M-MLV) reverse transcriptase (Invitrogen) according to manufacturer protocol. PCR sets were conducted in a 20 mL PCR mixture containing 12 pM of each forward and reverse primers, 1× reaction buffer including 35 µM MgCl_2_, 27 µM of each dNTP, 1.5 μL of cDNA, and 0.25 units of DNA polymerase (Thermo Fisher Scientific Inc., Waltham, MA, USA). The thermocycler was set up to denature DNA for 3 min at 94 °C, then go through 35 cycles of 94 °C for 40 s, 60 °C for 40 s, and 72 °C for 60 s, and a final extension step at 72 °C for 7 min. The primers listed in Table 3 were used for BMIV testing. PCR products were visualized in agarose gel (2%) stained with ethidium bromide.

## 4. Conclusions

This study was the first trial to eliminate BMIV from the sour black mulberry. Chemotherapy, thermotherapy, and cryotherapy, as well as their combinations, were applied to test their efficacy upon BMIV elimination. We developed a highly effective protocol by applying thermotherapy following chemotherapy using 30 mg/L ribavirin, which resulted in 50% efficiency for BMIV elimination from infected sour black mulberry. The results of this study may significantly contribute to producing virus-free propagative material, which can lead to safer germplasm exchange and higher income for farmers. Studies are to be continued to evaluate the performance of virus-free accession in the field conditions.

## Figures and Tables

**Table 1:** Comparison of survival rates of different treatments used in this study. The survival rate was calculated by dividing the number of surviving plants by the number of tested plants and multiplying by 100.

Treatment	Ribavirin Dose (mg/L)	No. of Cultured Plants	No. of Surviving Plants	Survival Rate (%)
Ch	0	60	45	75 ab*
	10	60	41	68.3 c
	20	60	35	58.3 cd
	30	60	36	60 cd
Ch + Th^1st^	0	36	28	77.8 ab
	10	36	25	69.4 bc
	20	36	27	75 ab
	30	36	25	69.4 bc
Th^2nd^ + Ch	0	36	35	97.2 ab
	10	36	36	100 a
	20	36	36	100 a
	30	36	36	100 a
Ch + PVS2	0	36	30	83.3 ab
	10	36	20	55.6 cd
	20	36	24	66.7 c
	30	36	25	69.4 bc
Cr	0	36	12	33.3 d

Ch: chemotherapy; Th: thermotherapy; PVS2: plant vitrification solution 2; Cr: cryotherapy * The same letter means no significant difference between treatments (*p* > 0.05).

**Table 2:** Efficacy of in vitro treatments for eliminating black mulberry idaeovirus (BMIV) applied to sour black mulberry.

Treatment	Ribavirin Dose (mg/L)	No. of Tested Plants	No. of Virus-Free Plants	Elimination Efficiency (%)
Ch	0	9	0	0
	10	7	0	0
	20	10	2	20
	30	9	3	33.3
Ch + Th^1st^	0	9	0	0
	10	9	0	0
	20	10	3	30
	30	10	5	50
Th^2nd^ + Ch	0	10	1	10
	10	10	2	20
	20	10	2	20
	30	10	2	20
Ch + PVS2	0	10	0	0
	10	10	0	0
	20	10	0	0
	30	10	0	0
Cr	0	10	0	0

Ch: chemotherapy; Th: thermotherapy; PVS2: plant vitrification solution2; Cr: cryotherapy.

**Table 3:** The list of the primers used to screen BMIV in the explants exposed to in vitro virus elimination treatments. The numbers in the primer name represent the nucleotide position of the primer on the BMIV RNA 1 genome.

Target	Primer	Sequence	Amplicon Size (bp)	Reference
Control	rbcL a	CTGCATGCATTGCACGGTG TACTTGAACGCTACTGCAG	186	[41]
	rbcL s			
BMIV	79F	CGGACTTTGTTGTTTGGAGTT CACCACTCTAATGGGGAAATG	282	[15]
	361R			
	362F	AAAAGAATGGGTTTAATAGCTCA TCATGTCTTCATCAGACAATTT	654	
	1016R			
	458F	TTGGATTGCGTCGGTGGTGGTTC TCATGTCTTCATCAGACAATTT	558	
	1016R			
	821F	TACGGTTGCTCCGTTTTCTCT GAAACAAACCGGTTTCAC	1295	
	2116R			
	1567F	GGTTCATTCCGGTTATGATTT	549	
	2116R	GAAACAAACCGGTTTCAC		
	1567F	GGTTCATTCCGGTTATGATTT CGTTTTCCAGAACAGTCATTTTT	946	
	2513R			
	1611F	GAATGGTCCAGCACGAAGTAA TCGAAGAAAACTGAGTCGTCA	982	
	2583R			
	3863F	GAAGCTAGTAAGTCCGAAGCTTCG	427	
	4290R	ACTTGCCTGCTGCTAGTATTTCTTCT		

## Data Availability

The datasets presented in the study are included in the article. The corresponding author can be contacted for further inquiries.

## Supplementary Materials

The following supporting information can be downloaded at https://www.mdpi.com/article/10.3390/plants13212959/s1, Figure S1: Agarose gel electrophoresis of RT-PCR products of nine samples cultured on 30 mg/L ribavirin using eight primer pairs targeting BMIV.
