# Supplementary material for: Efficacy of Plant Tissue Culture Techniques for Eliminating Black Mulberry Idaeovirus (BMIV) from Infected Black Mulberry (Morus nigra)
Source: Plants (Basel). 2024 Oct 23;13(21):2959. doi: 10.3390/plants13212959 (PMC11548174; doi:10.3390/plants13212959)
Supplement: Supplementary file 1 [file plants-13-02959-s001.zip › plants-3183639-supplementary.pdf]

Supplementary Figure S1

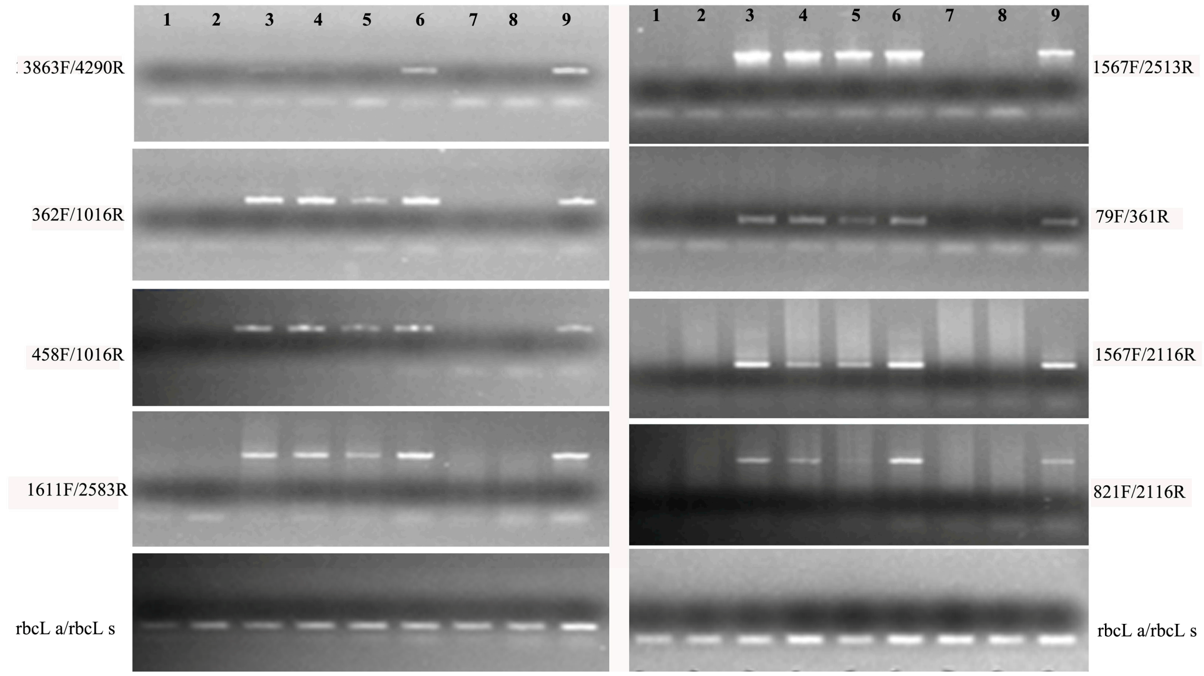

Figure S1. Agarose gel electrophoresis of RT-PCR products of nine samples cultured on 30mg/L ribavirin using eight primer pairs targeting BMIV. The Rbcl primers were used as internal control for RT-PCR assays. No amplification product was generated in lanes 1, 2, 7 and 8, and the corresponding explants were considered virus-free.
